# Cardiorespiratory Coupling Analysis Based on Entropy and Cross-Entropy in Distinguishing Different Depression Stages

**DOI:** 10.3389/fphys.2019.00359

**Published:** 2019-03-29

**Authors:** Lulu Zhao, Licai Yang, Zhonghua Su, Chengyu Liu

**Affiliations:** ^1^School of Control Science and Engineering, Shandong University, Jinan, China; ^2^Second Affiliated Hospital of Jining Medical College, Jining, China; ^3^School of Instrument Science and Engineering, Southeast University, Nanjing, China

**Keywords:** cardiorespiratory coupling, depression, sample entropy, fuzzy measure entropy, cross entropy, vagal modulation

## Abstract

**Aims:**

This study used entropy- and cross entropy-based methods to explore the cardiorespiratory coupling of depressive patients, and thus to assess the values of those entropy methods for identifying depression patients with different disease severities.

**Methods:**

Electrocardiogram (ECG) and respiration signals from 69 depression patients were recorded simultaneously for 5 min. Patients were classified into three groups according to the Hamilton Depression Rating Scale (HDRS) scores: group Non-De (HDRS 0–7), Mid-De (HDRS 8–17), and Con-De (HDRS >17). Sample entropy (SEn), fuzzy measure entropy (FMEn) and high-frequency power (HF) were computed on the original RR interval time series and breath-to-breath interval time series. Cross sample entropy (CSEn) and cross fuzzy measure entropy (CFMEn) were computed on interval time series resampled at 2 Hz and 4 Hz, respectively. The difference among three patient groups and correlation between entropy values and HDRS scores were analyzed by statistical analysis. Surrogate data were also employed to confirm the validation of entropy measures in this study.

**Results:**

A consistent increasing trend has been found among most entropy measures from Non-De, to Mid-De, and to Con-De groups, and a significant (*p* < 0.05) difference in FMEn of RR intervals exists between Non-De and Mid-De or Con-De groups. Significant differences have been also found in all cross entropies, between Non-De and Con-De groups and between Mid-De and Con-De groups. Furthermore, significant correlations also exist between HDRS scores and FMEn of RR intervals (*R* = 0.24, *p* < 0.05), CSEn at 4 Hz (*R* = 0.26, *p* < 0.05) or 2 Hz (*R* = 0.28, *p* < 0.05) resampling, and CFMEn at 4 Hz (*R* = 0.31, *p* < 0.01) or 2 Hz (*R* = 0.30, *p* < 0.05) resampling. A significant difference of cardiorespiratory coupling parameters between different depression stages and significant correlations between entropy measures and depression severity both indicate central autonomic dysregulation in depression patients and reflect varying degrees of vagal modulation reduction among different depression levels. Analysis based on surrogate data confirms that the non-linear properties of the physiological signals played a major role in depression recognition.

**Conclusion:**

The current study demonstrates the potential of cardiorespiratory coupling in the auxiliary diagnosis of depression based on the entropy method.

## Introduction

According to the World Health Organization, depression is the leading cause of disability worldwide and is a major contributor to the overall global burden of disease (WHO, 2018). The World Federation for Mental Health conducted a World Mental Health Survey in 17 countries, found that about 5% of people reported having depression in 2011 (WFMH, 2012). Except for the worst result suicide behavior, as a common mental disorder, depressions reduce the function of the human body and often are recurring. Furthermore, depression patients are found to have a higher-than-average risk of cardiovascular diseases and mortality (Zellweger et al., 2011). Unfortunately, the diagnosis of depression in clinical now is still subjective, depending on the psychiatrist’s personal judgment and experience, without any objective physiological data. Therefore, to explore the physiological characteristic of depression patients and find the quantitative relationship between depression and physiological data, turn out to be an extremely urgent and meaningful work.

Recently, central autonomic dysregulation has been suggested in patients with recurrent depression. On one hand, clinical symptoms such as sleep disturbances, dry mouth, and sweating support alterations in ANS activity in subjects suffering from depression (Chang et al., 2012). On the other hand, changed HRV, heart rate complexity and baroreflex sensitivity have been described in studies (Koschke et al., 2009; Schulz et al., 2010; Voss et al., 2011), indicating a reduction of vagal function in relation to the central autonomic dysregulation. Specifically, HRV has emerged as a physiological marker for emotional regulation (Shi et al., 2017), psychological well-being (Chalmers et al., 2014), and general cardiovascular health (Kemp and Quintana, 2013). Among the time-domain, frequency-domain and non-linear parameters, entropy is regarded as a valuable tool to quantify the regularity and inherent complexity of physiological time series and can provide important insights for understanding the underlying mechanisms of the cardiovascular and respiration systems (Zhao et al., 2015). Moreover, the human body is an organic whole system, and there are multifarious interactions among physiological systems. HRV as a measure acquired from the ECG might be affected by cardiovascular disease besides the ANS modulation. Therefore, non-linear measures of the coordination between heart rate and respiration have been recently introduced as useful indices of vagal output from the central autonomic network in subjects with a broad range of psychiatric disorders (Chang et al., 2012).

Heart rate variability indicates cardiac autonomic regulation, while the cardiorespiratory coupling measures the coupling between heart rate and respiration and reflects the two system’s association level controlled by the central autonomic network (Dick and Morris, 2004). Thus, by integrating HRV with the respiratory rhythm, the cardiorespiratory coupling can enhance the signal-to-noise ratio and can be used to evaluate the complex interactions between brainstem regions and higher regulatory centers (Chang et al., 2013). Different indices of cardiorespiratory coupling may present different aspects of the cardiorespiratory interaction from the HRV. The level of cardiorespiratory coupling is highly associated with the efferent vagal activity from the central autonomic network (Peupelmann et al., 2009). Cross-entropy measures were employed in this study to assess the cardiorespiratory coupling of depression patients.

To distinguish different pathophysiologic conditions of depression patients, this study employs SEn and FMEn to evaluate the regularity and complexity changes of ECG and respiration signals, as well as CSEn and CFMEn to investigate the strength difference of cardiorespiratory coupling among different severity depression patients. The frequency index, high-frequency power (HF, 0.15–0.40 Hz) is also measured, considering its role in reflecting the parasympathetic activity (O’Regan et al., 2015; Yeh et al., 2016). Reduced cardiorespiratory coupling, reflected by increasing cross entropy values, caused by reduced vagal modulation in central autonomic dysregulation was expected. Besides, to confirm that the expected differences in entropy values among different depressive severities are coming from the patients’ signals, instead of the random calculation errors, surrogate data of the physiological interval time series, as well as the difference sequences of physiological series and surrogate data were also analyzed.

## Materials and Methods

### Subjects

Sixty-nine patients were included in this study (48 females and 21 males, aging from 16 to 80). All of the patients were recruited from the Second Affiliated Hospital of Jining Medical College, Jining, Shandong, China. The protocol of this study was approved by the Ethics Committee of the Second Affiliated Hospital of Jining Medical College. All patients gave written informed consent in accordance with the Declaration of Helsinki. Patients were inpatient and were diagnosed by at least two staff psychiatrists, according to ICD-10 criteria for depression (International Classification of Diseases, 10th Edition). Complete medical and psychiatric histories of the patients were available through medical records. All patients were evaluated by the 17-item HDRS every week after their hospitalization (Hamilton, 1960), to assess the objective severity of depressive symptoms. And the score which was tested on the closest date to the electrophysiological signal acquisition date was recorded for each patient. According to the rating scale, for scores less than 7, the non-depressive state is concluded; for scores between 8 and 17, mild depression is suggested; for scores more than 17, depression is confirmed, and moderate or severe is estimated based on whether the score larger than 24 (Zhang, 2005). Therefore, the patients were divided into following three groups according to their HDRS scores: Non-De Group, including 15 patients with 0–7 scores, Mil-De Group, including 34 patients with 8–17 scores, and Con-De Group, including 20 patients with scores over 17. Table 1 shows the details of the demographic and clinical characteristics of the three groups.

**Table 1:** Demographic and clinical characteristics of the three groups.

	Group		
	Non-De	Mid-De	Con-De
No.	15	34	20
Gender, male/female	5/10	10/24	6/14
Age (year)	47 ± 15.88	42 ± 16.28	44 ± 14.54
Height (cm)	162.87 ± 6.47	163.85 ± 8.06	165.65 ± 7.61
Weight (kg)	66.73 ± 10.16	65.23 ± 12.24	67.8 ± 14.14
Education, ≤12 years/≥13 years	13/2	30/4	14/6
Occupation, yes/no	12/3	22/12	12/8
Right-handedness, yes/no	14/1	34/0^b^	17/3
Smoking, yes/no	3/12	0/34	3/17
Drinking, yes/no	1/14	0/34	0/20
Heart rate (beats/min)	82 ± 15.89	86 ± 16.09	85 ± 15.35
Breath rate (breaths/min)	16.16 ± 2.46	16.27 ± 2.94^b^	18.45 ± 2.97^a^
Systolic blood pressure (mmHg)	118 ± 10.99	112 ± 14.05	117 ± 19.22
Diastolic blood pressure (mmHg)	76 ± 9.06	71 ± 8.84	76 ± 13.62
Depression type, depression/bipolar disorder	13/2	31/3	16/4
HDRS score	3.93 ± 2.52	13.17 ± 3.00^aa,bb^	20.85 ± 2.20^aa^

*Data are expressed as number or mean ± standard deviation (std). ^a^Statistical significant difference with p < 0.05 when compared with correspondent data in group Non-De. ^aa^Statistical significant difference with p < 0.01 when compared with correspondent data in group Non-De. ^b^Statistical significant difference with p < 0.05 between group Mid-De and Con-De. ^bb^Statistical significant difference with p < 0.01 between group Mid-De and Con-de; otherwise, there is no significant difference.*

### Data Acquisition and Preprocessing

Data acquisition was executed in a sound attenuated and temperature-controlled suite located on the top floor of Inpatient Building, which had isolated waiting room and operating room, to avoid any interruption. Selected patients were made an appointment previously, and were taken to the suite from their wards accompanied by at least two medical staff in case of any emergency situation. A short-structured conversation was proceeded firstly, to record some clinical information, and to inform the whole process and matters needing attention. Then they were asked to keep supine position with eye closed, the whole body relaxed without any movement, and to calm down and breathe evenly.

**FIGURE 1:**
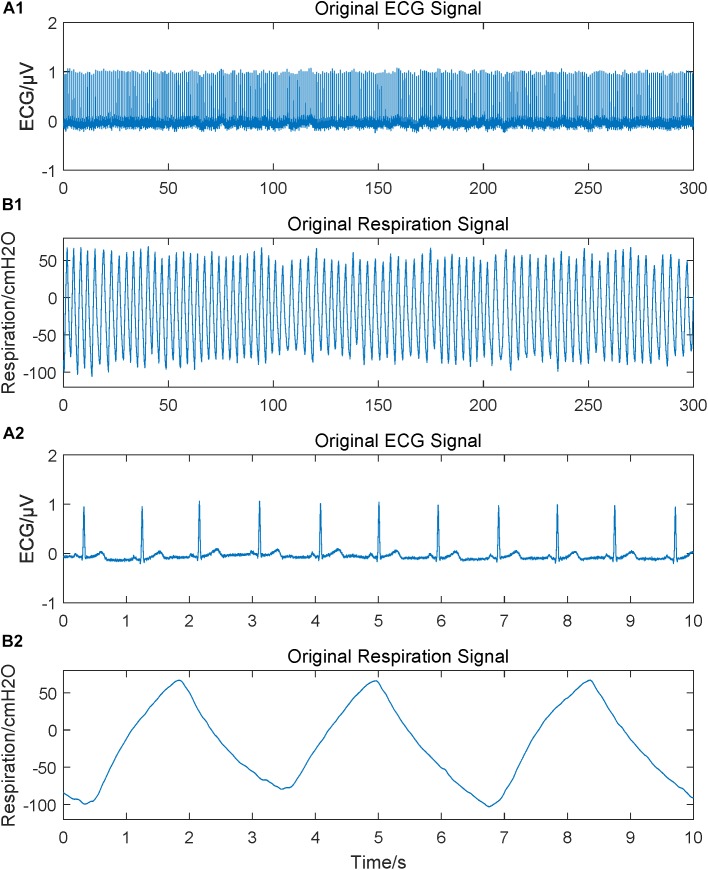
Original physiological signals obtained. Subgraph **A1** and **B1** are original ECG and respiration signals lasting for 5 min; **A2** and **B2** are 10 s segments to give a more intuitive and clearer waveform.

ECG and respiratory signals were recorded simultaneously at a sample rate of 1,000 Hz using a multichannel physiological acquisition system RM6280C (Chengdu Instrument Factory, Sichuan, China). Sensitivity parameters were set as 1 mV for ECG, and 50 cm H_2_O for respiration. Hardware filtering from the system was turned off to ensure the integrity of the valid signals. ECG signal was recorded using three electrodes placed on the right wrist and both ankles according to the standard limb lead-II ECG acquisition method, while the respiratory signal obtained by using an elastic abdominal belt with comfortable tightness. The recording started after both physiological signals had been smooth and steady and lasted for 5.5 min. Examples of the original ECG and respiration signals were shown in Figure 1. After the signal recording, heart rate and pulse pressure were measured using OMRON HEM-7051.

**FIGURE 2:**
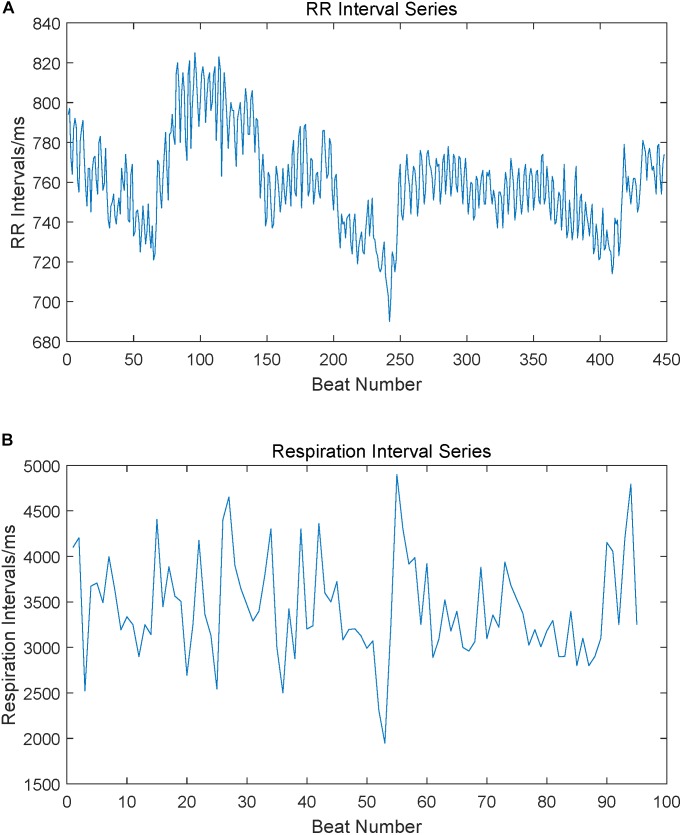
RR interval series and respiration peak interval series extracted from ECG and respiration signals. Subgraph **(A)** is a sample of the RR interval series extracted from ECG, subgraph **(B)** is a sample of the respiration peak interval series extracted from respiration signals.

Wavelet threshold filter was used on ECG signals to remove the noises and baseline drift, and second-order IIR peak filter was used over respiratory signals to increase the main frequency and to remove high-frequency interference (Li et al., 2017). Adaptive difference threshold method was used to extract the variability series, i.e., RR interval time series from ECG and peak-to-peak interval time series from respiratory signals (Shi et al., 2017). Both interval time series had been checked manually in case of omissive premature beats and the false detections from the automatic method had also been corrected manually. Although in some previous studies, respiration amplitude had been coupled into the RR intervals (Faes et al., 2015; Javorka et al., 2018), this study employed respiration interval time series to focus on the time rhythmicity, like the measures in RR intervals. Those variability series were filtered by an adaptive impulse rejection filter to replace and interpolate ventricular premature beats, heterogeneous breathing and other artifacts (Liu et al., 2012). Filtered variability series were shown in Figure 2. Finally, the filtered variability series were resampled into 4 Hz and 2 Hz, respectively, by using cubic spline interpolation, leading to a consistent frequency between ECG and respiratory signals (Liu et al., 2012). Considering the high degree of predictability of the cubic splines might affect the true complexity of the interval series, a time delay δ was used in cross entropy measures (Govindan et al., 2007). We used a 5-min variability series, which includes 1,200 sample points for 4 Hz resampling and 600 sample points for 2 Hz.

### Entropy Measure

Entropy-based measures, such as the typical approximate entropy (ApEn) and SEn, as a non-linear measurement to value the regularity of short physiological time series, have been widely used to explore their inherent complexity (Liu et al., 2015). Richman and Moorman (2000) proposed SEn since the ApEn produces biased estimation for the complexity of physiological signals with self-matching, which can be defined as the negative natural logarithm of the estimate of conditional entropy. By comparing the similarity of runs of patterns in time series, SEn is negatively correlated with the overall regularity, and a larger SEn value indicates a higher complexity of the time series, as well as a higher unpredictability (Hautala et al., 2010).

However, based on the Heaviside function, the SEn has a rigid boundary, which may induce to the non-uniform variation of the entropy value along with the change of the threshold *r* (Richman and Moorman, 2000). This was caused by the kernel estimator strategy employed, the Heaviside kernel function (Xiong et al., 2017). To avoid this limitation and to enhance the statistical stability, another kernel estimator, the fuzzy function was used to propose a fuzzy entropy, which produces a gradually varied entropy value with the changes of *r* (Chen et al., 2009; Xie et al., 2010a). But Liu and Zhao (2011) and Liu et al. (2013) found that fuzzy entropy overemphasized the local vector similarity and neglected the global characteristics, thus finally the novel FMEn was proposed to improve the discrimination ability. More details and definition of the FMEn could be found in the above references. Therefore, the typical SEn and the newly improved FMEn were employed in this study to calculate the regularity and complexity of the original RR intervals of ECG and breath-by-breath intervals of respiration signals.

### Cross-Entropy Measures

In recent years, it has been an increasing focus of interest to explore the synchronization and dynamic interaction between two distinct physiological signals by using cross-entropy measures (Xie et al., 2010b; Ahmed and Mandic, 2012; Li et al., 2013). According to the literature reading by far, two different methods exist in cross-entropy definition and calculating. Consider X and Y as two interacting time series. One cross-entropy is defined to value the possibility of one series could be predicted based on the information coming from the other series (Schulz et al., 2013; Widjaja et al., 2015). This measure pays more attention to the causal relationship from the past X to the present Y, with directivity, and ignore the effect coming from the past Y itself. The other cross entropy used in this study considers effect from both X and Y simultaneously and focuses more attention on the overall synchronism. Since this study aims to explore the influence of depression state to the asynchrony degree between ECG and respiration, the latter method was employed. The cross-entropy measures stated below are all based on the second definition. The cross-entropy evaluate interactions between two distinct but interacting time series (i.e., ECG and respiration in this study) under the influence of the central autonomic network (Chang et al., 2013), which is negatively correlated with the non-linear coupling level between two related series as it measures the asynchrony degree of two time series (Berger et al., 2010). It could be speculated that a strong association between RR intervals and respiration peak point intervals would lead to a small cross entropy value, indicating relatively high synchrony (Chang et al., 2012). In contrast, larger value cross entropy suggests a weaker association and lower synchrony. Based on the above discussion of SEn and FMEn, their generalized forms, CSEn and CFMEn were used in this study to test the synchronization between ECG and respiratory signals of depression patients. No matter for SEn and FMEn, or their generalized cross entropies, two unknown parameters, embedding dimension m and tolerance threshold r need to be initialized before performing entropy measures. According to recommended ranges were given by previous work, *m* between 1 and 3 (Zhao et al., 2015), and *r* between 0.1 and 0.25 (Pincus, 2010), and considering the short respiration interval series length in this study, a final combination of *m* = 2, *r* = 0.2 was employed. The value of time delay δ was equaled to the average cardiac circle, and after rounding off the actual value 1 was used in the calculation of cross entropies (Govindan et al., 2007).

### Surrogate Data Analysis

Surrogate data has been widely used to test the non-linearity of a time series in HRV (Porta et al., 2007; Eduardo Virgilio Silva and Otavio Murta, 2012), as it has the same linear properties and amplitude distribution compared with the original time series (Schreiber and Schmitz, 1996; Ocak, 2009), but the non-linear correlations has been destroyed (Schreiber and Schmitz, 1996; Ocak, 2009). A concise and efficient generation method based on Fourier Transform which was proposed by Schreiber and Schmitz (1999) was used in this study. Surrogate data of 69 subjects based on all six time intervals were generated separately, i.e., the original RR intervals and resampled RR intervals in 4 Hz and 2 Hz, as well as the original breath intervals and resampled breath intervals. All eight entropy measures and one frequency index had been calculated based on these surrogate data to compare with the original indexes values distribution. As the surrogate data has the same linear properties with the original time series, to confirm the effect of the non-linear properties in recognition depression severities reflected by entropy measures, we also employed a difference sequence, by calculating the difference between physiological series and surrogate data. Therefore, the entropy values of the difference sequence could be taken on behalf of the non-linear properties of patients signals.

### Statistical Analysis

Statistical analysis was conducted by employing MATLAB software (Ver. R2015b, MathWorks, United States). Eight entropy values, SEn of RR intervals (SEnRR), SEn of respiration intervals (SEnRes), FMEn of RR intervals (FMEnRR), FMEn of respiration intervals (FMEnRes), CSEn between RR and respiration intervals (including CSEn_4 based on 4 Hz resampling and CSEn_2 base on 2 Hz resampling), and CFMEn (including CFMEn_4 and CFMEn_2), as well as the frequency index, high frequency power (HF) were calculated among all 69 subjects, separately by the Non-De, Mid-De, Con-De groups. All indexes series were tested for normal distribution by Kolmogorov–Smirnov test firstly. If the index series passed the test, one-way ANOVA was used to test the difference among all three groups firstly, and then group *t*-test was used to test the statistical differences between every two groups. Otherwise, the Kruskal–Wallis rank test and Wilcoxon rank sum test were used for difference examination. The correlations between the above index values and HDRS scores were analyzed by Pearson correlation analysis. All statistical results were considered statistically significant with *p*-values less than 0.05.

## Results

According to the rating scale of HDRS, the subjects were divided into three groups representing three pathophysiologic conditions of depression, i.e., Non-De, Mil-De, and Con-De. As different severity of depression is suggested by HDRS scores, our results confirm this subjective evaluation to a certain extent by the changes in physiological signals, specialized in cardiorespiratory coupling analysis.

### Difference Significance

In difference significance analysis among all three groups, normal distribution test and variance homogeneity test were performed previously. Half of the entropy measures passed the test (SEnRR, CSEn_4, CFMEn_4, and CFMEn_2), therefore was tested by one-way ANOVA, while the other four entropy measures, as well as the frequency index which failed the test, were processed by Kruskal–Wallis rank test. The final difference results were shown in the first three columns in Table 2. For entropy measures based on the single physiological signal, either ECG or respiration, as well as the frequency indexes, no significance exists for both SEn and FMEn, while all cross entropy measures show significant difference among three groups.

**Table 2:** Index values and difference significance of physiological series with embedding dimension *m* = 2 and tolerance threshold *r* = 0.2.

	*F*	Chi-square	Non-De	Mid-De	Con-De
	(one-way ANOVA)	(Kruskal–Wallis rank test)	
SEnRR	1.87	–	1.50 ± 0.07	1.67 ± 0.05	1.61 ± 0.06
SEnRes	–	0.37	2.02 ± 0.13	1.97 ± 0.10	1.98 ± 0.08
FMEnRR	–	4.97	1.40 ± 0.10	1.64 ± 0.06^a^	1.66 ± 0.09^a^
FMEnRes	–	0.02	1.99 ± 0.09	2.00 ± 0.05	2.02 ± 0.06
CSEn_4	4.3^*^	–	0.61 ± 0.03	0.64 ± 0.02^b^	0.72 ± 0.03^a^
CFMEn_4	5.38^**^	–	0.36 ± 0.03	0.43 ± 0.02^b^	0.52 ± 0.04^aa^
CSEn_2	–	9.68^**^	1.10 ± 0.08	1.18 ± 0.05^bb^	1.47 ± 0.11^aa^
CFMEn_2	4.75^*^	–	0.81 ± 0.07	0.93 ± 0.02^b^	1.10 ± 0.08^a^
HF	–	2.37	152.94 ± 65.69	204.07 ± 41.09	220.33 ± 61.38

*Data are expressed as mean ± standard error (SE). ^∗^Statistical significance among all three groups with *p* < 0.05. ^∗∗^Statistical significance among all three groups with p < 0.01. ^a^Statistical significance compared with group Non-De with p < 0.05. ^aa^Statistical significance compared with group Non-De with p < 0.01. ^b^Statistical significance between group Mid-De and Con-De with p < 0.05. ^bb^Statistical significance between group Mid-De and Con-De with p < 0.01. SEnRR, sample entropy of RR interval series; SEnRes, sample entropy of respiration peak interval series; FMEnRR, fuzzy measure entropy of RR intervals; FMEnRes, fuzzy measure entropy of respiration peak intervals; CSEn_4 and CSEn_2, cross sample entropy of RR intervals and respiration peak intervals based on 4 Hz and 2 Hz resampling; CFMEn_4 and CFMEn_2, cross fuzzy measure entropy of RR intervals and respiration peak intervals based on 4 Hz and 2 Hz resampling; HF, high-frequency power of the original RR intervals.*

**FIGURE 3:**
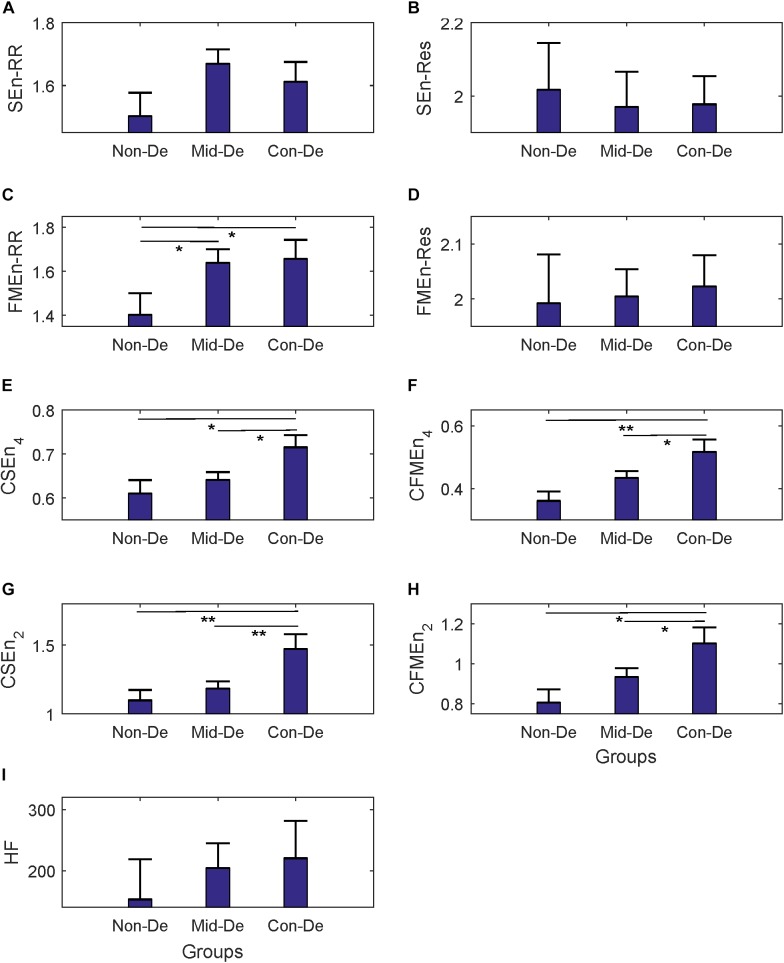
The frequency index and eight entropy measures’ distribution based on physiological series among three groups Non-De, Mid-De, and Con-De with embedding dimension *m* = 2 and tolerance threshold *r* = 0.2. **(A)** SEnRR, **(B)** SEnRes, **(C)** FMEnRR, **(D)** FMEnRes, **(E)** CSEn_4, **(F)** CFMEn_4, **(G)** CSEn_2, **(H)** CFMEn_2, **(I)** HF. The height of the bar indicates the mean entropy value of each group, with the length of the horizontal bar exceeding the main bar reflecting the standard error. ^∗^A significant difference between related two groups with *p* < 0.05. ^∗∗^A significant difference between related two groups with *p* < 0.01.

In difference significance analysis between every two groups, all eight entropy results were calculated with embedding dimension *m* = 2 and tolerance threshold *r* = 0.2, and are shown in the form of mean ± standard error in Table 2. Figure 3 shows relevant results distribution. Consistent increasing trends in both entropy and cross entropy were found with the increase of depression disease level as shown in Table 2, with only two exceptions, for the SEnRR average value of group Con-De had a decrease of 0.06 than Mid-De, and the SEnRes of group Mid-De had a decrease of 0.05 than Non-De. The increasing of entropy values from group Non-De to Mid-De, and from group Non-De to Con-De suggest a reduced regularity of the depressed patients, and a continue increasing unpredictability along with the depression deepens.

In the comparison between group Non-De and Mid-De, only the FMEnRR had a significant increase of 0.24 (*p* < 0.5) among all eight entropy measures while the others only showed a small increase in values. In comparison between group Non-De and Con-De, only FMEnRR showed a significant increase of 0.26 (*p* < 0.5) among all univariate sample entropies and fuzzy measure entropies. However, all cross entropies did have a significant increase. CFMEn_4 and CSEn_2 performed even better, with a significant increase of 0.16 and 0.37 (*p* < 0.01), while the other two only had significance with *p* < 0.05. By far, the developed algorithm discrimination ability of fuzzy kernel function based FMEn has been reflected compared with SEn, as discussed in Section “Entropy Measure.” In comparison between group Mid-De and Con-De, no significance in entropies based on single signal, while all cross measures had significant increases. CSEn_2 performs better (*p* < 0.01) than others (*p* < 0.05). For visually check the statistical differences, as shown in Figure 3, only the FMEnRR showed significant difference among all entropies based on the single signal, either ECG or respiration, while all cross measures showed a significant difference in both comparisons, between group Non-De and Con-De, and group Mid-De and Con-De. Consistent with the ANOVA results, cross measures based on both RR and respiratory intervals showed more powerful discrimination ability. The frequency index HF showed no significant difference among all group comparisons, although it did have a sustained growing trend, with an increase of 51.13 in group Mid-De than Non-De, and a continued increase of 16.26 in group Con-De. The large standard error (65.69 in Non-De, 41.09 in Mid-De, and 61.38 in Con-De) might be responsible for the weak distinguishability.

### Correlation and Regression Analysis

Table 3 showed the results of correlation analysis between index values based on physiological series and HDRS scores. Both difference analysis and correlation analysis results confirm that higher HDRS scores are accompanied with higher FMEnRR, CFMEn_4, CSEn_2, and CSEn_2 values, indicating increased unpredictability of both ECG and respiration signals, and decreased synchronization of cardiorespiratory coupling as well.

**Table 3:** Regression equations between nine indexes based on physiological series and HDRS scores.

Regression equation:	*R*-value	*R*^2^-value	*p*-Value
SEnRR = 0.0074 ∗ HDRS score + 1.5178	0.1735	0.0301	0.1539
SEnRes = -0.0013 ∗ HDRS score + 2.0002	-0.0181	0.0003	0.8825
FMEnRR = 0.0139 ∗ HDRS score + 1.4062	0.2406	0.0579	0.0465^*^
FMEnRes = -0.0006 ∗ HDRS score + 2.0147	-0.0132	0.0002	0.9141
CSEn_4 = 0.0047 ∗ HDRS score + 0.5933	0.2609	0.0681	0.0304^*^
CFMEn_4 = 0.0071 ∗ HDRS score + 0.3471	0.3138	0.0985	0.0086^**^
CSEn_2 = 0.0166 ∗ HDRS score + 1.0253	0.2822	0.0797	0.0188^*^
CFMEn_2 = 0.0138 ∗ HDRS score + 0.7704	0.3004	0.0902	0.0122^*^
HF = 5.6808 ∗ HDRS score + 121.5968	0.1494	0.0223	0.2206

*^∗^Significant correlation with p < 0.05. ^∗∗^Significant correlation with p < 0.01.*

Regression equations are listed in Table 3, which showed that only SEnRes (*R* = -0.0181) and FMEnRes (*R* = -0.0132) had negative relationships with HDRS scores, and all other seven measures had positive relationships with HDRS scores, although only FMEnRR, CSEn_4, CSEn_2, and CFMEn_2 had significance with *p* < 0.05, and CFMEn_4 had better significance (*p* < 0.01). Consistent with the significance analysis, the above five entropy measures also had higher correlation coefficient R, differentiate coefficients R square, and regression coefficient of the regression equation, which indicate that the HDRS can effectively predict the variation of corresponding entropy measures, compared with other three entropies, SEnRR, SEnRes, and FMEnRes. With especially higher coefficient values existed in CFMEn_4 and CFMEn_2, FMEn shows obvious advantages compared with SEn. However, most correlation coefficient values are quite small, even the highest value is below 0.4, which reflects a comparatively low linear correlation between HDRS score and related entropy measures.

### Surrogate Data Results

**FIGURE 4:**
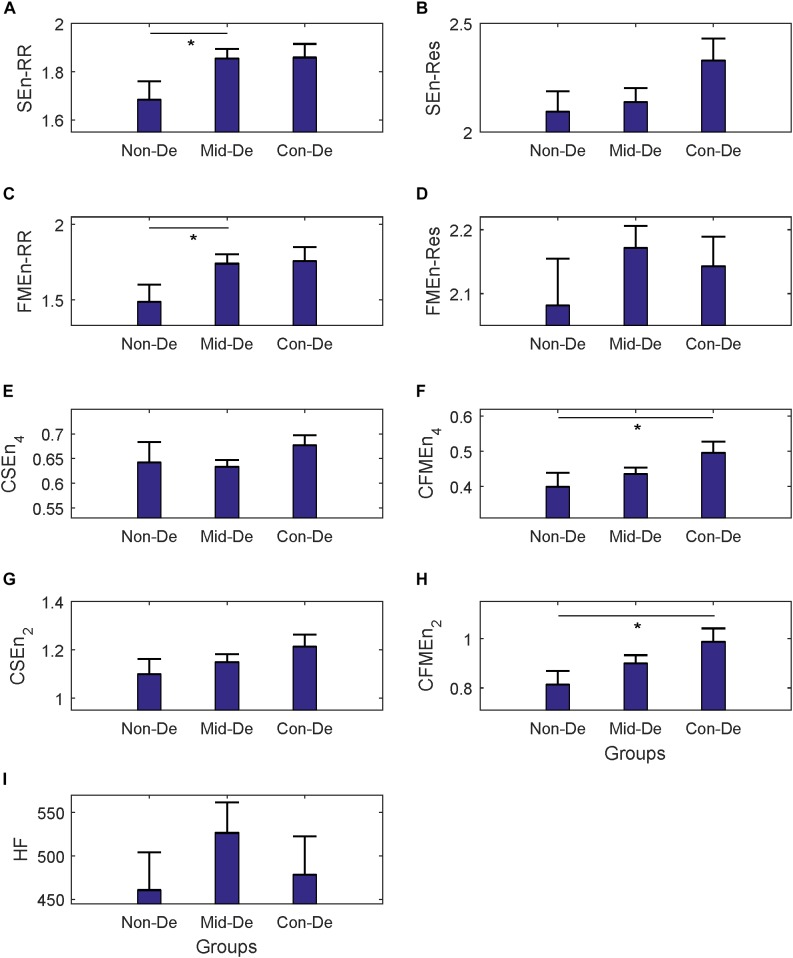
The frequency index and eight entropy measures’ distribution of surrogate data among three groups Non-De, Mid-De, and Con-De. **(A)** SEnRR, **(B)** SEnRes, **(C)** FMEnRR, **(D)** FMEnRes, **(E)** CSEn_4, **(F)** CFMEn_4, **(G)** CSEn_2, **(H)** CFMEn_2, **(I)** HF. The height of the bar indicates the mean entropy value of each group, with the length of the horizontal bar exceeding the main bar reflecting the standard error. ^∗^A significant difference between related two groups with *p* < 0.05. ^∗∗^A significant difference between related two groups with *p* < 0.01.

**FIGURE 5:**
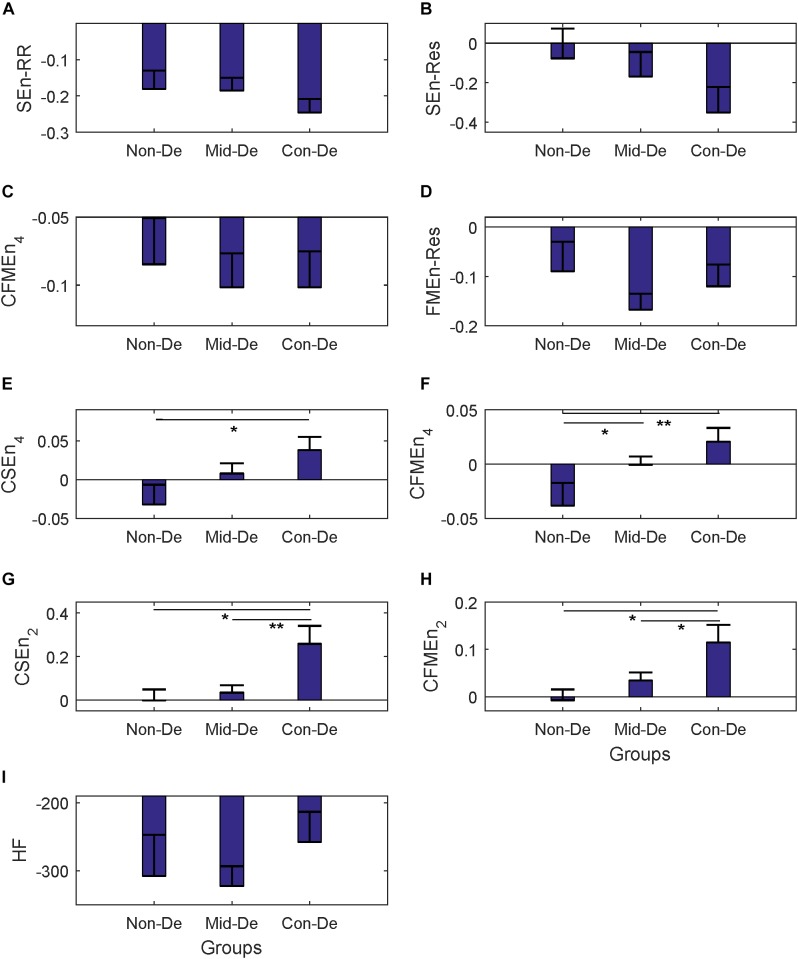
The frequency index and eight entropy measures’ distribution of difference sequences among three groups Non-De, Mid-De, and Con-De. **(A)** SEnRR, **(B)** SEnRes, **(C)** FMEnRR, **(D)** FMEnRes, **(E)** CSEn_4, **(F)** CFMEn_4, **(G)** CSEn_2, **(H)** CFMEn_2, **(I)** HF. The height of the bar indicates the mean entropy value of each group, with the length of the horizontal bar exceeding the main bar reflecting the standard error. ^∗^A significant difference between related two groups with *p* < 0.05. ^∗∗^A significant difference between related two groups with *p* < 0.01.

The same statistical analysis flow was performed on surrogate series and difference sequences separately, and the difference analysis results were listed in Table 4 and Figure 4 for surrogate series, and Table 5 and Figure 5 for difference sequences. The overall results based on surrogate data showed partly similar distribution trend compared with physiological time series, with only four indexes (FMEn_RR, CSEn_4, CFMEn_4, and CSEn_2) showed increasing trends among three groups, while the other five indexes showed different distributions with physiological series. The larger difference was reflected by the significance results. Much lower difference significance had been showed in surrogate data compared with physiological series as the significances existed in Figure 3 all disappeared in Figure 4, except for three ones, the significance in FMEn_RR between group Non-De and Mid-De, CFMEn_4 between Non-De and Con-De, and CFMEn_2 between Non-De and Con-De. However, the surrogate data also showed one more significance in SEn_RR between group Non-De and Mid-De which the physiological series did not have. Compared with surrogate data, the entropy distribution of difference sequences showed much more similar distribution trend to the physiological series. Especially for the cross entropy measures, the difference sequences showed almost the same significances except for only one less of CSEn_4 between group Mid-De and Con-De.

**Table 4:** Index values and difference significance based on surrogate data with embedding dimension *m* = 2 and tolerance threshold *r* = 0.2.

	*F*	Chi-square	Non-De	Mid-De	Con-De
	(one-way ANOVA)	(Kruskal–Wallis rank test)			
SEnRR	2.7	–	1.68 ± 0.08	1.85 ± 0.04^a^	1.86 ± 0.06
SEnRes	1.93	–	2.09 ± 0.09	2.14 ± 0.06	2.33 ± 0.10
FMEnRR	2.55	–	1.49 ± 0.12	1.74 ± 0.06^a^	1.76 ± 0.09
FMEnRes	–	1.59	2.08 ± 0.07	2.17 ± 0.03	2.14 ± 0.05
CSEn_4	–	5.88	0.64 ± 0.04	0.63 ± 0.01	0.68 ± 0.02
CFMEn_4	–	6.33^*^	0.40 ± 0.04	0.44 ± 0.02	0.50 ± 0.03^a^
CSEn_2	–	3.87	1.10 ± 0.06	1.15 ± 0.03	1.21 ± 0.05
CFMEn_2	2.92	–	0.81 ± 0.06	0.90 ± 0.03	0.99 ± 0.05^a^
HF	–	0.88	460.65 ± 43.53	526.42 ± 34.98	478.43 ± 43.93

*Data are expressed as mean ± standard error (SE). ^∗^Statistical significance among all three groups with p < 0.05. ^a^Statistical significance compared with group Non-De with p < 0.05.*

**Table 5:** Index values and difference significance based on difference sequences with embedding dimension *m* = 2 and tolerance threshold *r* = 0.2.

	*F*	Chi-square	Non-De	Mid-De	Con-De
	(one-way ANOVA)	(Kruskal–Wallis rank test)			
SEnRR	0.74	–	-0.18 ± 0.05	-0.19 ± 0.03	-0.25 ± 0.04
SEnRes	0.84	–	-0.08 ± 0.15	-0.17 ± 0.12	-0.35 ± 0.13
FMEnRR	–	1.1	-0.08 ± 0.03	-0.10 ± 0.03	-0.10 ± 0.03
FMEnRes	0.87	–	-0.09 ± 0.06	-0.17 ± 0.03	-0.12 ± 0.04
CSEn_4	3.15^*^	–	-0.03 ± 0.03	0.01 ± 0.01	0.04 ± 0.02^a^
CFMEn_4	–	8.13^*^	-0.04 ± 0.02	0.00 ± 0.01^a^	0.02 ± 0.01^aa^
CSEn_2	–	8.44^*^	0.00 ± 0.05	0.03 ± 0.03^bb^	0.26 ± 0.08^a^
CFMEn_2	–	5.96^*^	-0.01 ± 0.02	0.03 ± 0.02^b^	0.11 ± 0.04^a^
HF	0.71	–	-307.7160.46	-322.3428.88	-258.1044.68

*Data are expressed as mean ± standard error (SE). ^∗^Statistical significance among all three groups with p < 0.05. ^a^Statistical significance compared with group Non-De with p < 0.05. ^aa^Statistical significance compared with group Non-De with p < 0.01. ^b^Statistical significance between group Mid-De and Con-De with p < 0.05. ^bb^Statistical significance between group Mid-De and Con-De with p < 0.01.*

Based on surrogate data, the correlation analysis between index values and HDRS scores was shown in Table 6, and the regression equations were also listed in Table 6. Same results based on the difference sequences were shown in Table 7. Most correlation coefficient values in Table 6 showed decreased values, while most *R*-values in Table 7 had increased values. Besides, all correlation significances existed in Table 3 disappeared in Table 6, while Table 7 kept most significances with only one less in FMEnRR. The significance had even been improved in CFMEn_2, with *p* = 0.0045 in Table 7 and *p* = 0.0122 in Table 3. All the above results indicated much better correlations between entropy values and HDRS scores in difference sequences than surrogate data.

**Table 6:** Regression equations between nine indexes based on surrogate data and HDRS scores.

Regression equation:	*R*-value	*R*^2^-value	*p*-Value
SEnRR = 0.0088 ∗ HDRS score + 1.7007	0.2235	0.0500	0.0649
SEnRes = 0.0091 ∗ HDRS score + 2.0622	0.1486	0.0221	0.2230
FMEnRR = 0.0132 ∗ HDRS score + 1.5142	0.2141	0.0458	0.0774
FMEnRes = 0.0014 ∗ HDRS score + 2.1243	0.0428	0.0018	0.7271
CSEn_4 = 0.0009 ∗ HDRS score + 0.6364	0.0549	0.0030	0.6540
CFMEn_4 = 0.0038 ∗ HDRS score + 0.3941	0.1887	0.0356	0.1205
CSEn_2 = 0.0038 ∗ HDRS score + 1.1060	0.1160	0.0135	0.3426
CFMEn_2 = 0.0072 ∗ HDRS score + 0.8102	0.2172	0.0472	0.0730
HF = 2.3120 ∗ HDRS score + 467.2489	0.0785	0.0062	0.5212

**Table 7:** Regression equations between nine indexes based on difference sequences and HDRS scores.

Regression equation:	*R*-value	*R*^2^-value	*p*-Value
SEnRR = -0.0014 ∗ HDRS score – 0.1829	-0.0484	0.0023	0.6929
SEnRes = -0.0104 ∗ HDRS score – 0.0619	-0.1049	0.0110	0.3911
FMEnRR = 0.0007 ∗ HDRS score – 0.1080	0.0366	0.0013	0.7653
FMEnRes = -0.0020 ∗ HDRS score – 0.1096	-0.0663	0.0044	0.5885
CSEn_4 = 0.0038 ∗ HDRS score – 0.0431	0.2982	0.0889	0.0128^*^
CFMEn_4 = 0.0033 ∗ HDRS score – 0.0469	0.3577	0.1279	0.0025^**^
CSEn_2 = 0.0128 ∗ HDRS score – 0.0807	0.3055	0.0933	0.0107^*^
CFMEn_2 = 0.0066 ∗ HDRS score – 0.0398	0.3378	0.1141	0.0045^**^
HF = 3.3687 ∗ HDRS score – 345.6521	0.1155	0.0133	0.3448

*^∗^Significant correlation with p < 0.05. ^∗∗^Significant correlation with p < 0.01.*

## Discussion

This study presents evidence that variability analysis based on entropy measurement could offer a good way to monitor and evaluate depression since it is important to diagnose this disease objectively and accurately. Based on entropy measures, by analyzing HRV and respiratory rhythm variability of different depression levels, it is confirmed that consistent increasing trends in most entropy and cross entropy were found with the increase of depression disease levels, and the significant difference of entropy measures between two different depression levels was found, as well as the significant correlation between entropy values and depression levels. All the above results suggest an increased irregularity of heart rate and decreased cardiorespiratory coupling from group severely depressed patients compared with mild ones.

In the field of evaluating depression by cardiorespiratory signals, most studies used HRV index individually to distinguish depression patients from healthy normal people. By employing frequency domain features of HRV, Yeh et al. (2016) found the significant lower variance, the low-frequency band (Zellweger et al., 2011), the high-frequency band (HF), and a higher LF/HF ratio of unmedicated major depressive disorder (MDD) patients compared with healthy subjects. Besides, they found increased LF and HF in patients after agomelatine treatment and concluded that depression severity independently leads to the decreased HRV and vagal tone (Yeh et al., 2016). Chen et al. (2017) found lower LF, LF/HF, and refined composite multi-scale entropy of MDD patients compared with healthy controls in resting state, and even more other decreased HRV parameters in time, frequency and non-linear domain in other states, i.e., deep breath, Valsalva test, and standing up state. According to Kemp et al.’s (2010) review on impact of depression on HRV, decreased parameters including HF, standard deviations of normal-to-normal interval (SDNN) and root mean square successive difference (RMSSD), were exhibited by MDD patients, as well as increased LF/HF ratio, indicating an increased sympathetic activity and dysfunction ANS of MDD. Although reduced HRV measures have been reported in many studies, results of some specific parameters still have conflict, i.e., decreased LF was found in MDD patients by Yeh et al. (2016) and Chen et al. (2017), while no difference of LF was reported by Kemp et al. (2010); increased LF/HF was shown in Yeh and Kemp’s studies, while decreased LF/HF was found by Chen. In the study of O’Regan et al. (2015), influence of antidepressant medicine showed higher weight than the disease, as participants on antidepressants (both with or without depression) showed significant decreased HF compared with controls, while no significant difference had been found between depressive participants and non-depressive ones, no matter with or without antidepressants. In this study, only a sustained increasing trend of HF had been found along with the depression deepens without significant difference, which might due to the large standard error. In regard to entropy measures of HRV, increased FMEn (*p* < 0.05) in severe depression were found in this study, while reduced ApEn was reported by Berger et al. (2012). These inconsistencies may suggest that only HRV analysis is not valid enough to discriminate the changes induced by depression, as it is based on only one physiological signal and is easily influenced by complicated physical and medical conditions. However, different experimental design, especially different pharmacological treatments should also be responsible for the conflicts among different studies.

Therefore, respiratory measures and cardiorespiratory coupling method were put forward in this study, to explore a more powerful method for further quantifying the depression diagnose, and this improvement was also confirmed in our results. Compared with SEnRR, SEnRes, and FMEnRes, cross entropies based on ECG and respiration signals have better performance with larger group differences between each two groups, and larger correlation index R values. Especially for CFMEn_4, which has the largest R and is the only parameter has a significant correlation with *p* < 0.01, indicating a stronger correlation between CFMEn and depression severity. This better performance verifies the advantage of cardiorespiratory coupling method against HRV method based on single ECG signal, and the advantage of FMEn against traditional SEn as well. However, there were no difference significance nor correlation significance in SEnRes and FMEnRes, suggesting that single respiration signal is not valid enough to reflect the depression state, which might because that the short time series, 5 min, is not long enough for this comparatively low-frequency signals. Besides, non-stationarity in physiological time series has an influence on the conditional-based entropy calculation (Xiong et al., 2017). In this study, the stationarity of the employed time series was ensured from two aspects: (1) in the experiment progress, the patients had enough time for rest to make sure the stability of cardiovascular and respiration activities, to acquire the stable RR and respiration interval time series; (2) manually checked the detected the signal feature locations and manually excluded the potential premature beats to decrease the influence of the ectopic beats.

Physiology and pathology changes of depression patients can be speculated based on the above entropy measures and statistical analysis. HRV has been used widely as index to explore psychological well-being and general cardiovascular health (Chalmers et al., 2014), and decreased HRV had been reported by most studies when linear parameters were used (Koenig et al., 2016), indicating a disequilibrium between parasympathetic and sympathetic nervous systems (Thayer et al., 2012). The increased SEn and FMEn found in this study suggest a reduced regularity and an increased unpredictability of the depressed patients, as well as an irregularity of the ANS as well. Cross-entropy was used to measure the cardiorespiratory coupling, which is negatively correlated with the coupling level between ECG and respiration signals (Chang et al., 2013). According to our knowledge, Berger’s work is the first and only study investigating cardiorespiratory coupling in unmedicated MDD patients by employing cross-approximate entropy, and larger entropy values were found in patients compared with healthy controls, however, without statistical significance (Berger et al., 2012). By employing a different strategy in measuring cross entropy between RR and respiration, Widjaja et al. (2015) explored the cardiorespiratory information dynamics during mental arithmetic and sustained attention, and no difference in cardiorespiratory coupling was found when several mental states were compared. In our study, with improved algorithm, significant increased CSEn and CFMEn of group Con-De compared with Non-De or Mid-De were shown in Figure 3, illustrating that depression may lead to decreased synchronicity between ECG and respiration signals, and the development of the disease could deepen these synchronization decrease. Different findings might due to different methodological, as Berger used cross-approximate entropy, while Widjaja employed a totally different measuring strategy in cross entropy. Because the network interaction between heart rate and respiration is mainly governed by vagal modulation (Berger et al., 2010), reduced cardiorespiratory coupling appears a decrease in the efferent vagal activity from the central autonomic network. Besides, there was also a significant increase of the breath rate in group Con-De compared with both Non-De (an increase of 2.29 with *p* < 0.05) and Mid-De (an increase of 2.18 with *p* < 0.05), as shown in Table 1. This significant higher breath rate could also suggest the central autonomic dysregulation due to lower parasympathetic activity since the parasympathetic inputs should have enabled the biological system to respond flexibly to environmental changes (Chang et al., 2012).

From the above results of surrogate data, it could be summarized that there is a changed distribution of surrogate data compared with the physiological series, and a very similar distribution of the difference sequences to the physiological series. Besides, there is a much better correlation between entropy values and HDRS scores in difference sequences than surrogate data. All the above points confirmed that the discriminability of depression severities is contributed by the patient’s signals, instead of the random calculation errors, and the non-linear properties of the signals have played a major role. However, since it is the entropy values of physiological series which showed the most significances between different depression groups and the best correlation between entropy values and HDRS scores, the linear properties also have a certain effect on depression discrimination.

This current study has several limitations. First of all, the size of the patient group is small, and there is a lack of healthy control group since the subjects all come from hospitalization patients, the control group consists of normal subjects should be taken in the following study. Secondly, the influence of different pharmacological treatments was not recorded in detail in this research, leading to a miss of the elimination of the effect of drug action on experimental results. Besides, by employing the peak-to-peak interval time series of respiration, the breath volume information has been ignored, another strategy in cardiorespiratory coupling mentioned in Section “Materials and Methods” could be compared in future, as well as another measure strategy of cross entropy mentioned in section “Cross-Entropy Measures.” Finally, although the entropy measures showed discriminability between different depressive severities, analysis based on surrogate data indicated that linear component also could distinguish different depressive groups, therefore more linear indexes should be explored to find the best index in depression recognition.

## Conclusion

In conclusion, the current study explored the changes in the complexity of RR and respiration intervals, as well as the cardiorespiratory coupling along with the depression level increases, by applying the entropy and cross-entropy methods. The differences of eight entropy measures between groups have been studied, and the relevance between HDRS scores and depression levels has been analyzed. The current results demonstrate that in patients with depression or bipolar disorder patients with a depressive episode, the values of cardiorespiratory coupling between heart rate and respiration, reflected by CSEn and CFMEn, are closely associated with depression severity. Significant differences among different groups and significant correlations between entropy values and depression level, both confirm the positive correlation relationship between two above cross entropy values and depression severity. Analysis of surrogate data confirmed both linear and non-linear properties of patients’ physiological signal have been influenced by depression. Subjects with higher HDRS scores have higher cross entropy values, indicating a higher level of asynchronization between ECG and respiration deal to the low vagal modulation, which might be caused by the effect of depression on the central autonomic network. It is a potential and significant method by measuring cardiorespiratory coupling to help to diagnose and to assess depressive severity.

## Data Availability

The datasets generated for this study are available on request to the corresponding author.

## Author Contributions

LZ, CL, and LY conceived the study. LZ and ZS collected the data. CL performed the model construction. LZ contributed to the data process and analysis and wrote the manuscript. All authors reviewed and approved the manuscript, and are in agreement with its main findings and conclusions.

## Conflict of Interest Statement

The authors declare that the research was conducted in the absence of any commercial or financial relationships that could be construed as a potential conflict of interest.

## Abbreviations

ANS: autonomic nervous system
CFMEn: cross fuzzy measure entropy
CSEn: cross sample entropy
ECG: electrocardiogram
FMEn: fuzzy measure entropy
FMEnRes: fuzzy measure entropy of respiration peak intervals
FMEnRR: fuzzy measure entropy of RR intervals
HDRS: Hamilton Depression Rating Scales
HRV: heart rate variability
ICD-10: International Classification of Diseases
10th Edition: RR, R point of ECG to R point of ECG
SEn: sample entropy
SEnRes: sample entropy of respiration peak intervals
SEnRR: sample entropy of RR intervals

## References

[B1] Ahmed M. U. Mandic D. P. (2012). Multivariate multiscale entropy analysis. *IEEE Signal Process. Lett.* 19 91–94. 10.1109/lsp.2011.2180713

[B2] Berger S. Boettger M. K. Tancer M. Guinjoan S. M. Yeragani V. K. Bar K. J. (2010). Reduced cardio-respiratory coupling indicates suppression of vagal activity in healthy relatives of patients with schizophrenia. *Prog. Neuropsychopharmacol. Biol. Psychiatry* 34 406–411. 10.1016/j.pnpbp.2010.01.009 20083149

[B3] Berger S. Kliem A. Yeragani V. Bar K. J. (2012). Cardio-respiratory coupling in untreated patients with major depression. *J. Affect Disord.* 139 166–171. 10.1016/j.jad.2012.01.035 22386048

[B4] Chalmers J. A. Quintana D. S. Abbott M. J. Kemp A. H. (2014). Anxiety disorders are associated with reduced heart rate variability: a meta-analysis. *Front. Psychiatry* 5:80. 10.3389/fpsyt.2014.00080 25071612 PMC4092363

[B5] Chang J. S. Ha K. Yoon I. Y. Yoo C. S. Yi S. H. Her J. Y. (2012). Patterns of cardiorespiratory coordination in young women with recurrent major depressive disorder treated with escitalopram or venlafaxine. *Prog. Neuropsychopharmacol. Biol. Psychiatry* 39 136–142. 10.1016/j.pnpbp.2012.06.002 22699029

[B6] Chang J. S. Lee S. D. Ju G. Kim J. W. Ha K. Yoon I. Y. (2013). Enhanced cardiorespiratory coupling in patients with obstructive sleep apnea following continuous positive airway pressure treatment. *Sleep Med.* 14 1132–1138. 10.1016/j.sleep.2013.04.024 24051114

[B7] Chen W. Zhuang J. Yu W. Wang Z. (2009). Measuring complexity using fuzzyEn, apEn, and sampEn. *Med. Eng. Phys.* 31 61–68. 10.1016/j.medengphy.2008.04.005 18538625

[B8] Chen X. Yang R. Kuang D. Zhang L. Lv R. Huang X. (2017). Heart rate variability in patients with major depression disorder during a clinical autonomic test. *Psychiatry Res.* 256 207–211. 10.1016/j.psychres.2017.06.041 28646783

[B9] Dick T. E. Morris K. F. (2004). Quantitative analysis of cardiovascular modulation in respiratory neural activity. *J. Physiol.* 556(Pt 3), 959–970. 10.1113/jphysiol.2003.060418 14978205 PMC1664997

[B10] Eduardo Virgilio Silva L. Otavio Murta L. (2012). Evaluation of physiologic complexity in time series using generalized sample entropy and surrogate data analysis. *Chaos* 22:043105. 10.1063/1.4758815 23278040

[B11] Faes L. Porta A. Nollo G. (2015). Information decomposition in bivariate systems: theory and application to cardiorespiratory dynamics. *Entropy* 17 277–303. 10.3390/e17010277

[B12] Govindan R. B. Wilson J. D. Eswaran H. Lowery C. L. Preißl H. (2007). Revisiting sample entropy analysis. *Phys. A Stat. Mech. Appl.* 376 158–164. 10.1016/j.physa.2006.10.077

[B13] Hamilton M. (1960). A rating scale for depression. *J. Neurol. Neurosurg. Psychiatry* 23 56–62. 10.1136/jnnp.23.1.56 14399272 PMC495331

[B14] Hautala A. J. Karjalainen J. Kiviniemi A. M. Kinnunen H. Mäkikallio T. H. Huikuri H. V. (2010). Physical activity and heart rate variability measured simultaneously during waking hours. *Am. J. Physiol. Heart Circ. Physiol.* 298 H874–H880. 10.1152/ajpheart.00856.2009 20023121

[B15] Javorka M. Krohova J. Czippelova B. Turianikova Z. Lazarova Z. Wiszt R. (2018). Towards understanding the complexity of cardiovascular oscillations: Insights from information theory. *Comput. Biol. Med.* 98 48–57. 10.1016/j.compbiomed.2018.05.007 29763765

[B16] Kemp A. H. Quintana D. S. (2013). The relationship between mental and physical health: insights from the study of heart rate variability. *Int. J. Psychophysiol.* 89 288–296. 10.1016/j.ijpsycho.2013.06.018 23797149

[B17] Kemp A. H. Quintana D. S. Gray M. A. Felmingham K. L. Brown K. Gatt J. M. (2010). Impact of depression and antidepressant treatment on heart rate variability: a review and meta-analysis. *Biol. Psychiatry* 67 1067–1074. 10.1016/j.biopsych.2009.12.012 20138254

[B18] Koenig J. Kemp A. H. Beauchaine T. P. Thayer J. F. Kaess M. (2016). Depression and resting state heart rate variability in children and adolescents - a systematic review and meta-analysis. *Clin. Psychol. Rev.* 46 136–150. 10.1016/j.cpr.2016.04.013 27185312

[B19] Koschke M. Boettger M. K. Schulz S. Berger S. Terhaar J. Voss A. (2009). Autonomy of autonomic dysfunction in major depression. *Psychosom. Med.* 71 852–860. 10.1097/PSY.0b013e3181b8bb7a 19779146

[B20] Li F. Yang L. Shi H. Liu C. (2017). Differences in photoplethysmography morphological features and feature time series between two opposite emotions: Happiness and sadness. *Artery Res.* 18 7–13. 10.1016/j.artres.2017.02.003

[B21] Li P. Liu C. Wang X. Li L. Yang L. Chen Y. (2013). Testing pattern synchronization in coupled systems through different entropy-based measures. *Med. Biol. Eng. Comput.* 51 581–591. 10.1007/s11517-012-1028-z 23337958

[B22] Liu C. Li K. Zhao L. Liu F. Zheng D. Liu C. (2013). Analysis of heart rate variability using fuzzy measure entropy. *Comput. Biol. Med.* 43 100–108. 10.1016/j.compbiomed.2012.11.005 23273774

[B23] Liu C. Li L. Zhao L. Zheng D. Li P. Liu C. (2012). A combination method of improved impulse rejection filter and template matching for identification of anomalous intervals in RR sequences. *J. Med. Biol. Eng.* 32 245–249. 10.5405/jmbe.1006

[B24] Liu C. Zhang C. Zhang L. Zhao L. Liu C. Wang H. (2015). Measuring synchronization in coupled simulation and coupled cardiovascular time series: a comparison of different cross entropy measures. *Biomed. Signal Process. Control* 21 49–57. 10.1016/j.bspc.2015.05.005

[B25] Liu C. Zhao L. (2011). “Using Fuzzy Measure Entropy to improve the stability of traditional entropy measures,” in *Proceedings of Computing in Cardiology*, Hangzhou, 681–684.

[B26] Ocak H. (2009). Automatic detection of epileptic seizures in EEG using discrete wavelet transform and approximate entropy. *Expert Syst. Appl.* 36 2027–2036. 10.1016/j.eswa.2007.12.065

[B27] O’Regan C. Kenny R. A. Cronin H. Finucane C. Kearney P. M. (2015). Antidepressants strongly influence the relationship between depression and heart rate variability: findings from the irish longitudinal study on ageing (TILDA). *Psychol. Med.* 45 623–636. 10.1017/S0033291714001767 25075912 PMC4413849

[B28] Peupelmann J. Boettger M. K. Ruhland C. Berger S. Ramachandraiah C. T. Yeragani V. K. (2009). Cardio-respiratory coupling indicates suppression of vagal activity in acute schizophrenia. *Schizophr. Res.* 112 153–157. 10.1016/j.schres.2009.03.042 19406623

[B29] Pincus S. M. (2010). Assessing serial irregularity and its implications for health. *Ann. N. Y. Acad. Sci.* 954 245–267. 10.1111/j.1749-6632.2001.tb02755.x 11797860

[B30] Porta A. Guzzetti S. Furlan R. Gnecchi-Ruscone T. Montano N. Malliani A. (2007). Complexity and nonlinearity in short-term heart period variability: comparison of methods based on local nonlinear prediction. *IEEE Trans. Biomed. Eng.* 54 94–106. 10.1109/TBME.2006.883789 17260860

[B31] Richman J. S. Moorman J. R. (2000). Physiological time-series analysis using approximate entropy and sample entropy. *Am. J. Physiol. Heart Circ. Physiol.* 278:H2039. 10.1152/ajpheart.2000.278.6.H2039 10843903

[B32] Schreiber T. Schmitz A. (1996). Improved surrogate data for nonlinearity tests. *Phys. Rev. Lett.* 77:635. 10.1103/PhysRevLett.77.635 10062864

[B33] Schreiber T. Schmitz A. (1999). Surrogate time series. *Phys. D Nonlinear Phenomena* 142 346–382. 10.1016/S0167-2789(00)00043-9

[B34] Schulz S. Adochiei F. C. Edu I. R. Schroeder R. Costin H. Bar K. J. (2013). Cardiovascular and cardiorespiratory coupling analyses: a review. *Philos. Trans. A Math. Phys. Eng. Sci.* 371:20120191. 10.1098/rsta.2012.0191 23858490

[B35] Schulz S. Koschke M. Bär K.-J. Voss A. (2010). The altered complexity of cardiovascular regulation in depressed patients. *Physiol. Measure.* 31 303–321. 10.1088/0967-3334/31/3/003 20086275

[B36] Shi H. Yang L. Zhao L. Su Z. Mao X. Zhang L. (2017). Differences of heart rate variability between happiness and sadness emotion states: a pilot study. *J. Med. Biol. Eng.* 37 527–539. 10.1007/s40846-017-0238-0

[B37] Thayer J. F. Ahs F. Fredrikson M. Sollers J. J. III Wager T. D. (2012). A meta-analysis of heart rate variability and neuroimaging studies: implications for heart rate variability as a marker of stress and health. *Neurosci. Biobehav. Rev.* 36 747–756. 10.1016/j.neubiorev.2011.11.009 22178086

[B38] Voss A. Boettger M. K. Schulz S. Gross K. Bar K. J. (2011). Gender-dependent impact of major depression on autonomic cardiovascular modulation. *Prog. Neuropsychopharmacol. Biol. Psychiatry* 35 1131–1138. 10.1016/j.pnpbp.2011.03.015 21453741

[B39] WFMH (2012). *DEPRESSION A Global Crisis.* Available at: http://www.who.int/mental_health/management/depression/wfmh_paper_depression_wmhd_2012.pdf (accessed 9 20, 2018).

[B40] WHO (2018). *Depression.* Available at: http://www.who.int/news-room/fact-sheets/detail/depression (accessed 9 20, 2018).

[B41] Widjaja D. Montalto A. Vlemincx E. Marinazzo D. Van Huffel S. Faes L. (2015). Cardiorespiratory information dynamics during mental arithmetic and sustained attention. *PLoS One* 10:e0129112. 10.1371/journal.pone.0129112 26042824 PMC4456404

[B42] Xie H. B. Guo J. Y. Zheng Y. P. (2010a). A comparative study of pattern synchronization detection between neural signals using different cross-entropy measures. *Biol. Cybern.* 102 123–135. 10.1007/s00422-009-0354-1 20033208

[B43] Xie H.-B. Zheng Y.-P. Guo J.-Y. Chen X. (2010b). Cross-fuzzy entropy: a new method to test pattern synchrony of bivariate time series. *Inform. Sci.* 180 1715–1724. 10.1016/j.ins.2010.01.004

[B44] Xiong W. Faes L. Ivanov P. C. (2017). Entropy measures, entropy estimators, and their performance in quantifying complex dynamics: effects of artifacts, nonstationarity, and long-range correlations. *Phys. Rev. E* 95:062114. 10.1103/PhysRevE.95.062114 28709192 PMC6117159

[B45] Yeh T. C. Kao L. C. Tzeng N. S. Kuo T. B. Huang S. Y. Chang C. C. (2016). Heart rate variability in major depressive disorder and after antidepressant treatment with agomelatine and paroxetine: findings from the taiwan study of depression and anxiety (TAISDA). *Prog. Neuropsychopharmacol. Biol. Psychiatry* 64 60–67. 10.1016/j.pnpbp.2015.07.007 26216863

[B46] Zellweger M. J. Osterwalder R. H. Langewitz W. Pfisterer M. E. (2011). Coronary artery disease and depression. *Nihon Rinsho* 69 (Suppl. 7):604.

[B47] Zhang Z. (2005). *Behavioral Medicine Scale Manual.* Beijing: China medical electronic audio and video publishing house.

[B48] Zhao L. Wei S. Zhang C. Zhang Y. Jiang X. Liu F. (2015). Determination of sample entropy and fuzzy measure entropy parameters for distinguishing congestive heart failure from normal sinus rhythm subjects. *Entropy* 17 6270–6288. 10.3390/e17096270

